# Supplementary material for: Effects of the Interface between Inorganic and Organic Components in a Bi2Te3–Polypyrrole Bulk Composite on Its Thermoelectric Performance
Source: Materials (Basel). 2021 Jun 4;14(11):3080. doi: 10.3390/ma14113080 (PMC8200231; doi:10.3390/ma14113080)
Supplement: Supplementary file 1 [file materials-14-03080-s001.zip › materials-1222276-supplementary.pdf]

materials

MDPI

Supplementary Materials

Effects of the interface between inorganic and organic components in a Bi_2Te_3-polypyrrole bulk composite on its thermoelectric performance

Cham Kim [1,*] and David Humberto Lopez [2]

[1] Daegu Gyeongbuk Institute of Science and Technology (DGIST), 333 Techno Jungang-daero, Daegu, 42988, Republic of Korea; charming0207@dgist.ac.kr
[2] Department of Chemical and Environmental Engineering, University of Arizona, 1133 E. James. E. Rogers Way, Tucson, AZ, 85721, USA; davidlopez3@email.arizona.edu
* Correspondence: charming0207@dgist.ac.kr

Physical density data

Table S1. Different physical densities of the pristine Bi_2Te_3 (BT) and Bi_2Te_3-polypyrrole (BT-PPY) specimens sintered at 350 and 400 °C. Relative densities of the BT specimens were given by comparing to the theoretical density of Bi_2Te_3 (7.86 g cm^{-3}).

	BT_350	BT_400	BT-PPY_350	BT-PPY_400
Physical density / g cm^{-3}	7.69	7.73	7.32	7.34
Relative density / %	97.8	98.3	-	-

Citation: Lastname, F.; Lastname, F.; Lastname, F. Title. *Materials* **2021**, *14*, 3080. https://doi.org/10.3390/ma14113080

Academic Editor: Jong-Soo Rhyee

Received: 29 April 2021
Accepted: 31 May 2021
Published: 4 June 2021

Publisher's Note: MDPI stays neutral with regard to jurisdictional claims in published maps and institutional affiliations.

Copyright: © 2021 by the authors. Licensee MDPI, Basel, Switzerland. This article is an open access article distributed under the terms and conditions of the Creative Commons Attribution (CC BY) license (http://creativecommons.org/licenses/by/4.0/).

Lattice parameters

Table S2. Lattice parameters of the pristine Bi_2Te_3 (BT) and Bi_2Te_3-polypyrrole (BT-PPY) sintered at 350 and 400 °C.

	BT_350	BT_400	BT-PPY_350	BT-PPY_400
a / Å	4.3426	4.3425	4.3427	4.3427
c / Å	30.4396	30.4385	30.4393	30.4386

The lattice parameters were calculated on the basis of the XRD results (Figure 2) using the following relation [1]. Combination of the Bragg's law and plane-spacing equation gives the relation below,

$$\sin^2\theta = \frac{\lambda^2}{4}\left[\frac{4}{3}\left(\frac{h^2+hk+k^2}{a^2}\right)+\frac{l^2}{c^2}\right]$$

where θ is the diffraction angle, a and c are lattice constants, *hkl* are miller indices for the crystal planes. $\lambda^2/4$ has a value of 0.594 Å^2 for Cu K_α radiation.

Specific heat (C_p)

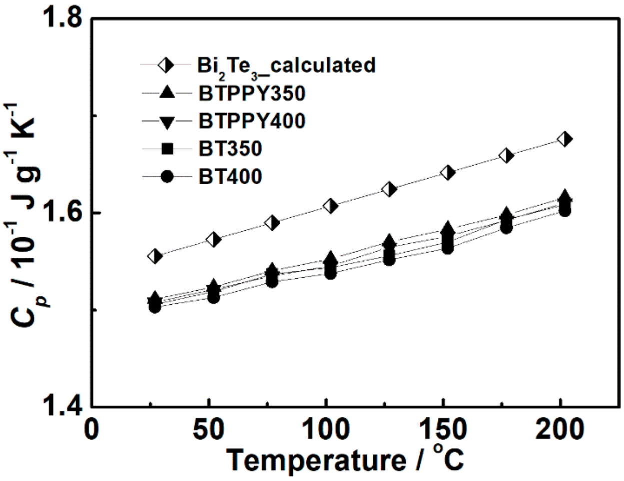

Figure S1. Temperature dependence of specific heat capacity (C_p) of the pristine Bi_2Te_3 and Bi_2Te_3-polypyrrole specimens sintered at 350 and 400 °C. The empirical polynomial equation, $C_p = a + b$T, describing the behavior of C_p as a function of temperature, was also used to derive the temperature dependence of C_p for Bi_2Te_3 [2]. The measured and calculated C_p values were in close proximity, with a deviation of less than 5% throughout the temperature range studied.

In-situ data of spark plasma sintering process

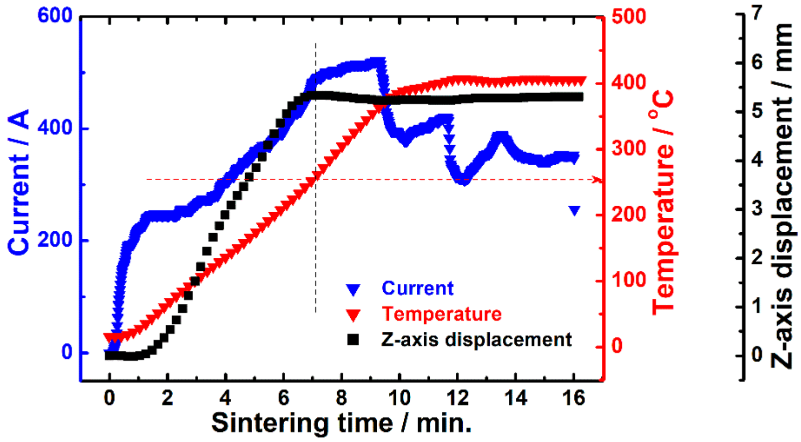

Figure S2. Variation in the Z-axis displacement of a graphite mold filled with the Bi_2Te_3-polypyrrole during the spark plasma sintering process at 400 °C, which was accompanied by the control of electric current.

X-ray diffraction (XRD) data

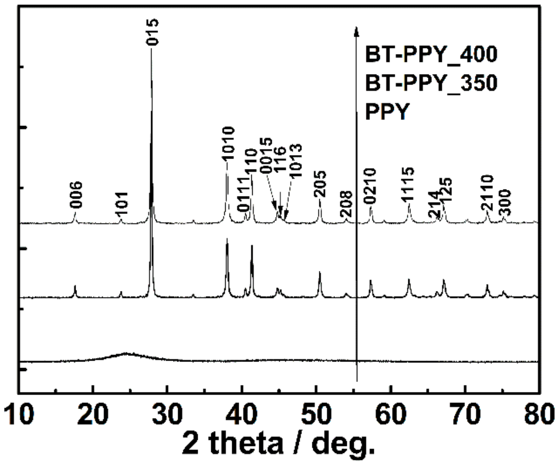

Figure S3. XRD pattern of polypyrrole, which was compared to that of the Bi_2Te_3-polypyrrole (BT-PPY) sintered at 350 or 400 °C.

Transmission electron microscopy (TEM)

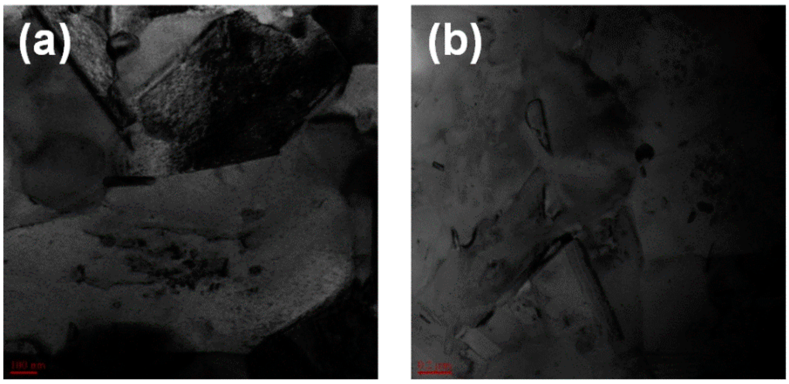

Figure S4. TEM images of the pristine Bi_2Te_3 specimens sintered at 350 (a) and 400 °C (b).

Thermoelectric properties

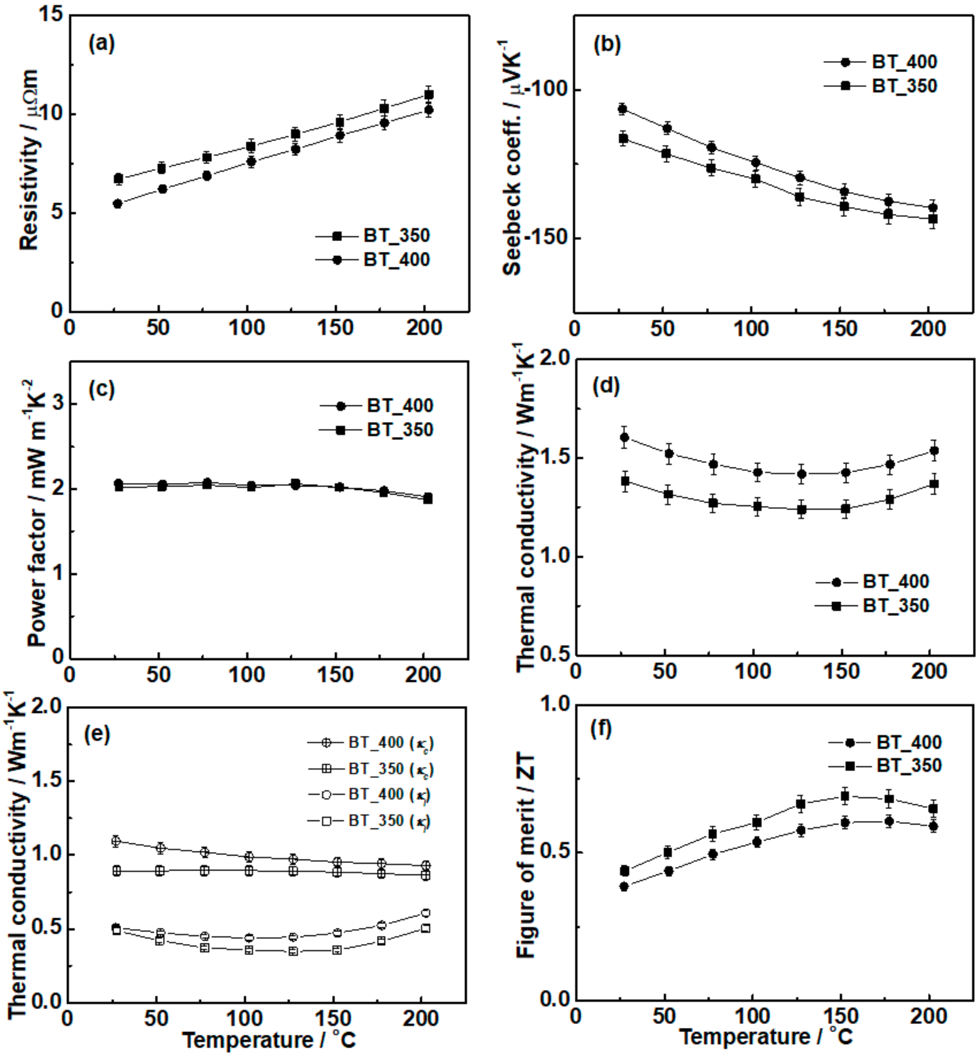

Figure S5. Thermoelectric transport properties of the pristine Bi_2Te_3 specimens sintered at 350 and 400 °C: (a) electrical resistivity, (b) Seebeck coefficient, (c) power factor, (d) thermal conductivity divided into (e) carrier (κ_c) and lattice (κ_l) contributions, and (f) ZT. Error bars are shown when they exceed the symbol size.

As the sintering temperature increased, the pristine Bi_2Te_3 exhibited the increase in carrier concentration (Table S3), resulting in the decreases in both electrical resistivity and Seebeck coefficient (Figure S5a, S5b). The electrical properties were offset, thus affording no considerable variation in the power factor (Figure S5c). The pristine Bi_2Te_3 exhibited the increased thermal conductivity with the elevated sintering temperature because both carrier and lattice thermal contributions increased (Figure S5d, S5e). The increased carrier and lattice contributions may result from the increased carrier concentration and possible grain growth, respectively. Because of the variation in thermal conductivity rather than electrical properties, the pristine Bi_2Te_3 sintered at 350 °C recorded higher ZT values than that sintered at 400 °C (Figure S5f).

Carrier concentration and mobility

Table S3. Carrier concentration (n) and mobility (μ) of the pristine Bi_2Te_3 (BT) and Bi_2Te_3-polypyrrole (BT-PPY) specimens sintered at 350 and 400 °C.

	BT_350	BT_400	BT-PPY_350	BT-PPY_400
n / 10^{19} cm^{-3}	5.02	5.61	3.91	4.16
μ / cm^2 V^{-1} s^{-1}	185	203	148	161

ZT values

Table S4. ZT values (ZT maximum: ZT_{max}, and ZT average: ZT_{ave}) of the Bi_2Te_3-polypyrrole compared to those of n-type binary Bi_2Te_3 and ternary $Bi_2(Te,Se)_3$ previously reported.

	**Present work**	**Bi_2Te_3 [3]**	**$Bi_2Te_{2.5}Se_{0.5}$ [4]**	**$Bi_2Te_{2.7}Se_{0.3}$ [5]**
ZT_{max}	1.18 (100 °C)	1.16 (150 °C)	1.18 (190 °C)	1.10 (120 °C)
ZT_{ave} (50–150 °C)	1.12	1.00	0.94	0.93

The Bi_2Te_3-polypyrrole recorded the ZT maximum (ZT_{max}) and average (ZT_{ave}) values of 1.18 at 100 °C and 1.12 at 50–150 °C, respectively. Compared to the recently reported n-type equivalents, the Bi_2Te_3-polypyrrole exhibited the ZT_{max} value at relatively low temperature. In addition, the Bi_2Te_3-polypyrrole was confirmed to exhibit higher ZT_{ave} value than the equivalents by 12–20%. Due to the superior ZT values at low temperatures below 150 °C, the Bi_2Te_3-polypyrrole is expected to be highly applicable to the promising thermoelectric operations at low temperatures, such as energy harvesting devices and systems.

References (only for the Supplementary Materials)

1. Hammond, C. *The Basics of Crystallography and Diffraction*; Oxford University Press: New York, 2001.
2. Binnewies, M.; Milke, E. *Thermochemical Data of Elements and Compounds*; Wiley-VCH: Weinheim, 1999.
3. Wu, F.; Song, H.; Gao, F.; Shi, W.; Jia, J.; Hu, X. *J. Electron. Mater.* **2013**, *42*, 1140–1145.
4. Xu, B.; Feng, T.; Agne, M.T.; Zhou, L.; Ruan, X.; Snyder, G.J.; Wu, Y. *Angew. Chem.* **2017**, *56*, 3546–3551.
5. Hong, M.; Chasapis, T.; Chen, Z.G.; Yang, L.; Kanatzidis, M.G.; Snyder, G.J.; Zou, J. *ACS Nano* **2016**, *10*, 4719–4727.
